# Outlier analyses to test for local adaptation to breeding grounds in a migratory arctic seabird

**DOI:** 10.1002/ece3.2819

**Published:** 2017-03-12

**Authors:** Anna Tigano, Allison J. Shultz, Scott V. Edwards, Gregory J. Robertson, Vicki L. Friesen

**Affiliations:** ^1^ Department of BiologyQueen's UniversityKingstonONCanada; ^2^ Department of Organismic and Evolutionary Biology and Museum of Comparative ZoologyHarvard UniversityCambridgeMAUSA; ^3^ Environment and Climate Change CanadaMount PearlNLCanada

**Keywords:** Arctic, genome assembly, outlier SNPs, population genomics, RADseq, thick‐billed murre, *Uria lomvia*

## Abstract

Investigating the extent (or the existence) of local adaptation is crucial to understanding how populations adapt. When experiments or fitness measurements are difficult or impossible to perform in natural populations, genomic techniques allow us to investigate local adaptation through the comparison of allele frequencies and outlier loci along environmental clines. The thick‐billed murre (*Uria lomvia*) is a highly philopatric colonial arctic seabird that occupies a significant environmental gradient, shows marked phenotypic differences among colonies, and has large effective population sizes. To test whether thick‐billed murres from five colonies along the eastern Canadian Arctic coast show genomic signatures of local adaptation to their breeding grounds, we analyzed geographic variation in genome‐wide markers mapped to a newly assembled thick‐billed murre reference genome. We used outlier analyses to detect loci putatively under selection, and clustering analyses to investigate patterns of differentiation based on 2220 genomewide single nucleotide polymorphisms (SNPs) and 137 outlier SNPs. We found no evidence of population structure among colonies using all loci but found population structure based on outliers only, where birds from the two northernmost colonies (Minarets and Prince Leopold) grouped with birds from the southernmost colony (Gannet), and birds from Coats and Akpatok were distinct from all other colonies. Although results from our analyses did not support local adaptation along the latitudinal cline of breeding colonies, outlier loci grouped birds from different colonies according to their non‐breeding distributions, suggesting that outliers may be informative about adaptation and/or demographic connectivity associated with their migration patterns or nonbreeding grounds.

## 1 Introduction

Natural selection is multidimensional in that selective forces can vary both spatially and temporally. For example, conspecific populations that are exposed to different environments (e.g., along clines) can experience different selection regimes. Individuals can also experience different selective pressures within a lifetime across different environmental patches and different seasons. In migratory species, conditions at the breeding vs. non‐breeding grounds can be very different and expose organisms to different sources of mortality. If “local habitat” is defined as the conditions at a given point in time and space (Kawecki & Ebert, 2004), do breeding or non‐breeding local habitats exert the stronger selection on migratory species? Atlantic salmon (*Salmo salar*) populations from different rivers, for example, are adapted to local conditions at natal sites (Dionne, Caron, Dodson, & Bernatchez, 2008; Dionne, Miller, Dodson, Caron, & Bernatchez, 2007). The conditions experienced during reproduction and early growth could exert the strongest selection on a population and define the local habitat to which populations are adapted.

Although the most definitive tests of local adaptation require common‐garden or reciprocal transplant experiments and fitness estimates to partition environment and genetic effects (Barrett & Hoekstra, 2011; Savolainen, Lascoux, & Merilä, 2013), these experiments are often difficult to perform due to logistics, ethics, and the life history characteristics of many species. An alternative approach to study local adaptation in natural populations is to compare phenotypic differences or allele frequencies along environmental gradients or across heterogeneous environments (Edwards, Shultz, & Campbell‐Staton, 2015; Savolainen et al., 2013). For example, clinal variations in color in the European barn owl (*Tyto alba*) and tawny owl (*Strix aluco*) seem to be maintained by environmental selection (Antoniazza, Burri, Fumagalli, Goudet, & Roulin, 2010; Karell, Ahola, Karstinen, Valkama, & Brommer, 2011), and clinal variation in allele frequencies in functional genes has been associated with various environmental factors, such as altitude (Bonin, Taberlet, Miaud, & Pompanon, 2006; McCracken et al., 2009), latitude (de Jong, Collins, Beldade, Brakefield, & Zwaan, 2013), and photoperiod (Bradshaw & Holzapfel, 2008). In recent years, several studies have highlighted the utility of genome‐wide scans to investigate mechanisms of population divergence and local adaptation (e.g., resolution of species boundaries in Lake Victoria cichlids, Wagner et al., 2013; parallel adaptation in salmonid fishes, Miller et al., 2012; burrowing behavior in oldfield mice *Peromyscus polionotus*, Weber, Peterson, & Hoekstra, 2013). An exponential increase in the number of genotyped loci and coverage of the genome over previous methods has also increased power to detect loci that deviate from a neutral model of evolution (i.e., outlier loci) and potentially underlie adaptation. Outlier analyses have allowed researchers to detect loci putatively under selection, which were then able to reveal fine differentiation patterns in otherwise homogenous populations and species (Hess, Campbell, Close, Docker, & Narum, 2013; Keller et al., 2013; Milano et al., 2014).

The thick‐billed murre (*Uria lomvia*) provides a useful model for studying local adaptation. It is a long‐lived colonial seabird with a wide distribution in arctic and subarctic regions of the Northern Hemisphere. In the summer, during the breeding season, thick‐billed murres nest on coastal cliffs. After chicks fledge, thick‐billed murres migrate south toward ice‐free areas. As a migratory species with a wide distribution, thick‐billed murres experience spatial and temporal variation in environmental conditions. We hypothesize that thick‐billed murres are adapted to local conditions at the breeding grounds because selection should act most strongly during the breeding time due to (1) the energy demands of breeding, (2) high overwinter survival (Smith & Gaston, 2012), and (3) a correlation between breeding success and environmental conditions around the colony during the breeding period at the Coats colony (Smith & Gaston, 2012).

Several studies and observations of morphological and behavioral differences support our hypothesis. Murres from different colonies differ in body size (Gaston, Chapdelaine, & Noble, 1984; Nettleship & Birkhead, 1985): Murres from Akpatok Island, for example, are significantly smaller than murres from other colonies (Gaston et al., 1984). Diet differs among breeding colonies in relation to local environmental conditions, especially sea ice (Provencher, Gaston, O'Hara, & Gilchrist, 2012). Birds from different colonies also differ in migration routes, timing, strategies, and wintering grounds (Gaston et al., 2011; McFarlane Tranquilla et al., 2013).

Thick‐billed murres are also exposed to environmental differences at their breeding areas, including differences in photoperiod, air and sea surface temperature, and timing of spring ice breakup and fall freeze‐up (see Appendix S1). Some of these environmental factors have documented fitness effects, either direct or indirect. Sea ice, for example, can impact murres’ fitness in several ways. Timing of sea ice breakup and freeze‐up affects breeding phenology, thus directly affecting reproductive success (Gaston, Gilchrist, & Hipfner, 2005). Sea ice affects migration strategies and routes because murres do not fly over ice (or land), and different migration strategies likely have different energy requirements (Gaston et al., 2011). Sea ice extent affects prey availability (Provencher et al., 2012). Temperature also affects murres’ survival: A combination of warm days and mosquito attacks caused dehydration and heat stroke in murres from Coats Island in some years (Gaston, Hipfner, & Campbell, 2002).

Significant adaptive differences among thick‐billed murres from different colonies are likely, and not only because their range spans a significant environmental gradient. Adaptation is predicted to evolve faster in large populations compared to small ones (Lanfear, Kokko, & Eyre‐Walker, 2014), and murres have large genetically effective population sizes (Birt‐Friesen, Montevecchi, Gaston, & Davidson, 1992). Gene flow can hamper adaptation (reviewed in Tigano & Friesen, 2016), but gene flow among thick‐billed murres from different colonies is probably very low because of their strong philopatry (Gaston, DeForest, Donaldson, & Noble, 1994; Steiner & Gaston, 2005). However, previous studies based on mitochondrial and microsatellite markers indicated that population structure is lacking within the Atlantic Ocean in thick‐billed murres, despite their philopatric behavior (Birt‐Friesen et al., 1992; Tigano et al., 2015). Additionally, thick‐billed murres presumably occupied their current range in the Atlantic since the retreat of the Pleistocene glaciers <10,000–15,000 years ago (Tigano et al., 2015). Therefore, thick‐billed murres provide a good system to investigate local adaptation because the factors that might confound the signatures of selection in the genome, such as genetic drift or population structure, are minimized, and the time of divergence between colonies is known.

In this study, we used double‐digest restriction‐site‐associated DNA sequencing (ddRADseq; Peterson, Weber, Kay, Fisher, & Hoekstra, 2012) and outlier analyses to test whether thick‐billed murres adapted to local conditions at their breeding grounds within the past 10,000–15,000 years, and investigated whether adaptive variation, if present, is clinal along the latitudinal range. To align and map RAD markers and thus improve accuracy in locus building and variant calling, we assembled a draft reference genome of the thick‐billed murre. To identify the genetic basis of phenotypic differences among colonies is not the aim of our study. Rather, we seek to investigate whether selection occurs during the breeding period, and hypothesize that patterns of differentiation among colonies are consistent with local adaptation at the breeding grounds. From a conservation perspective, estimating levels of differentiation and standing genetic variation will help us to understand the potential of thick‐billed murres to adapt to future environmental change.

## 2 Methods

### 2.1 Sample collection and DNA extraction

**Figure 1:**
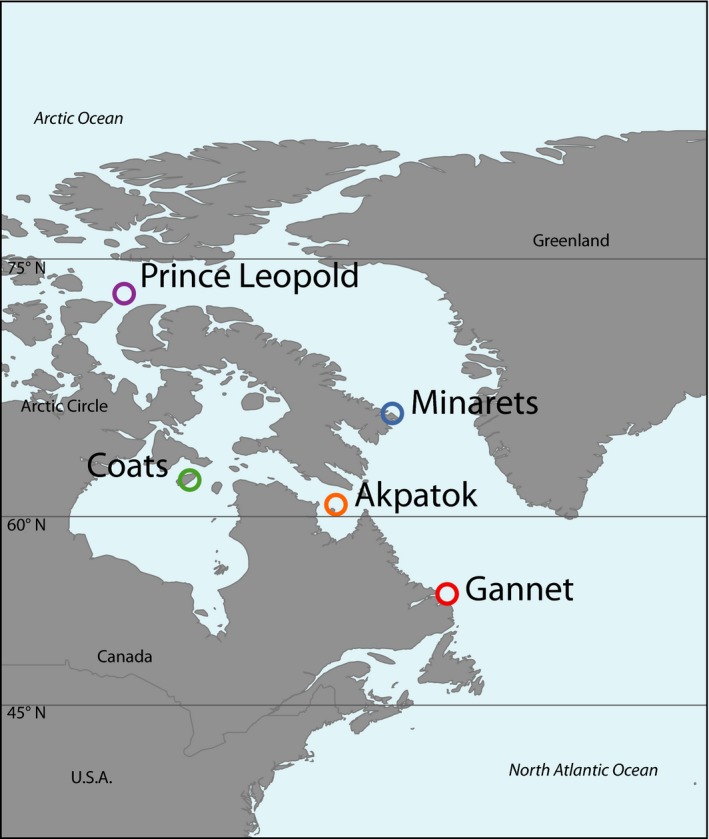
Map showing colony locations

Among the colonies distributed along the Canadian Atlantic coast, we selected five colonies along a latitudinal cline that includes birds from the extremes of the distribution (Gannet and Prince Leopold, ~20° latitudinal difference), known migratory behavior (Gannet, Coats, Minarets, Prince Leopold), and morphometric distinctiveness (Akpatok; Figure 1). We used previously collected blood samples from breeding adults from each colony (Birt‐Friesen et al., 1992; Tigano et al., 2015; Environment and Climate Change Tissue Archive; Figure 1, Table 1). Samples were collected from breeding adults from each colony during June‐July in 1987 (Akpatok), 1996 (Coats and Gannet), and 2008 (Minarets and Prince Leopold) and stored in ethanol at −80°C. We also included samples of four common murres (*Uria aalgae*), the sister species of the thick‐billed murre, as an out group to test for the presence of hybrids (Taylor, Patirana, Birt, Piatt, & Friesen, 2012). We purified DNA for library preparation using standard protease k/phenol–chloroform extraction protocols and ethanol precipitation with resuspension in DNase‐free water (Sambrook, Fritsch, & Maniatis, 1989).

**Table 1:** Sampling sites, initial and (final) sample size (*n*), geographical coordinates, and abbreviations

Colony	*n*	Latitude	Longitude	Abbreviation
Gannet Islands	18 (15)	53°42′N	56°12′W	Gannet
Akpatok Island	20 (19)	60°32′N	68°32′W	Akpatok
Coats Island	19 (14)	62°57′N	82°00′W	Coats
The Minarets (Baffin Island)	20 (19)	66°56′N	61°44′W	Minarets
Prince Leopold Island	19 (19)	74°01′N	90°02′W	Prince Leopold

### 2.2 De novo genome assembly

We sequenced the whole genome of a female thick‐billed murre from Coats Island. Genomic libraries were prepared and sequenced at the Genome Quebec Innovation Centre (McGill, Montreal). Short‐insert paired‐end libraries were prepared using Illumina TruSeq DNA Sample Prep Kit v2 targeting inserts of 320 bp. To improve scaffolding, long‐insert libraries of three different insert sizes (2, 5, and 10 kb) were prepared with Illumina Nextera Mate Pair Sample Preparation Kit. All libraries were sequenced in three lanes of Illumina HiSeq2500 with 100 bp paired‐end sequencing. We trimmed short‐insert paired‐end reads from the 3′ end until a minimum phred score of 30 was reached, filtered for a minimum read length of 50, and adapter‐clipped using trimmomatic v.0.30 (Bolger, Lohse, & Usadel, 2014). The fraction of paired reads surviving the trimming step was above 95% for all three read sets. We preprocessed long‐insert paired‐end reads using nextclip v.0.7 (Leggett, Clavijo, Clissold, Clark, & Caccamo, 2014). Only read pairs that contained the adaptor in at least one of the reads were retained. The proportion of usable pairs was between 34 and 36% for all read sets. We assembled the preprocessed reads to create a first set of consensus sequences using the assembler implemented in ray v.2.3.0 (Boisvert, Laviolette, & Corbeil, 2010). After reviewing assembly statistics and coverage statistics for various k‐mer sizes, *K *=* *31 was chosen for the definitive assembly. Contigs were merged in scaffolds with sspace v.3.0 (Boetzer, Henkel, Jansen, Butler, & Pirovano, 2011), using the reads from the mate‐pair libraries. We calculated the k‐mer coverage distribution and inferred mean base coverage using the following formula:

$$c_{\mathrm{base}}=c_{k-\mathrm{mer}}\times L/\left(L-K+1\right)$$

where *c*_base_ is the base coverage, *c*_k‐mer_ is the k‐mer coverage, *L* is the read length, and *K* is the k‐mer size.

### 2.3 RAD library preparation

We prepared RAD libraries using the ddRADseq protocol as described in Peterson et al. (2012). Briefly, we digested 500 ng of DNA from each sample using the restriction enzymes *SpH1* and *EcoR1*. We ligated each sample to adapters barcoded with a set of eight sequences (8 bp long) that differed by at least two base positions to maximize assignment probability. We pooled uniquely barcoded samples and extracted 300 to 350 bp fragments using a Pippin Prep (Sage Science, Inc., Beverly, MA, USA). To add Illumina indices with a unique sequence for each pooled library, and to increase concentrations of sequencing libraries, we amplified a total of 26–190 ng of pooled, size‐selected DNA using high‐fidelity PCR for 16 cycles (KAPA HotStart Long Range PCR Kit; KAPA Biosystems, Wilmington, MA, USA). We cleaned PCR products with AMPure beads (Beckman Coulter, Inc., Brea, CA, USA) and pooled libraries in equimolar amounts. We sequenced 93 samples using one lane of Illumina HiSeq2500 paired‐end 150 bp sequencing from a rapid run flow cell at the Genome Quebec Innovation Centre.

### 2.4 Variant calling

We used geneious v.6.1.5 (http://www.geneious.com, Kearse et al., 2012) to demultiplex raw sequence reads and trim the restriction‐site sequence at the 5′ end of each read. We then processed the demultiplexed reads using the program process_radtags in stacks v.1.06 (Catchen, Amores, Hohenlohe, Cresko, & Postlethwait, 2011; Catchen, Hohenlohe, Bassham, Amores, & Cresko, 2013) to remove sequences with either low‐quality scores or uncalled bases. We aligned processed reads to the thick‐billed murre reference genome using bowtie2 v.2.1.0 (Langmead & Salzberg, 2012) and discarded reads that had more than one match to the genome. We analyzed aligned reads using the programs ref_map.pl and populations in stacks (Catchen et al., 2011, 2013). We called SNPs using a maximum‐likelihood statistical model implemented in ref_map.pl requiring a minimum of two identical reads to create a stack. We generated input files (structure and genepop format) for downstream analyses with the program populations. Loci included in the final dataset had at least 10× depth of sequencing and were present in at least 90% of the individuals in each of the five colonies included in this study. To test for presence of common murre X thick‐billed murre hybrids, we repeated the analysis including the four samples of common murres with the same settings. This dataset included only one SNP per locus, retrieved in stacks using the option “–write_single_snp.”

To assess differentiation among colonies and levels of genetic diversity, we calculated global and population diversity and *F*‐statistics for each SNP using genodive (Meirmans & Van Tienderen, 2004) and stacks, respectively. Major allele frequencies, percentage of polymorphic sites, and π indicate the general level of genetic diversity in a population. *F*_IS_ is the inbreeding coefficient, and positive values indicate nonrandom mating or cryptic population structure. *F*_ST_ is a measure of population differentiation based on differences in allele frequencies.

### 2.5 Outlier analyses

We applied further filtering to our SNP dataset by discarding loci that had minor allele frequencies <5% in at least one population to minimize detection of false positives in subsequent outlier analyses, and we intersected lists of loci from each colony to minimize the occurrence of missing values in the final dataset. Then, we created 10 subsets of data representing each of the 10 possible colony pairs. To detect loci putatively under selection, we performed outlier analyses for each pairwise comparison using two different approaches: the Bayesian method implemented in the program bayescan v.2.1 (Foll & Gaggiotti, 2008) and the Fdist2 program (Beaumont & Nichols, 1996) implemented in lositan (Antao, Lopes, Lopes, Beja‐Pereira, & Luikart, 2008). bayescan is more conservative and less prone to false positives than lositan, but its statistical power decreases with the number of populations and with weak selection, while lositan provides a more robust method than bayescan when the number of populations screened is low, or when populations deviate from the island model of migration on which lositan is based (Mita et al., 2013; Narum & Hess, 2011). bayescan uses a logistic regression model to partition *F*_ST_ coefficients into a population‐specific component (beta) and a locus‐specific component (alpha). A positive value of alpha at a given locus suggests positive selection. Estimated model parameters were obtained by running the analysis using 20 pilot runs, each consisting of 5,000 iterations, followed by 100,000 iterations with a burn‐in of 50,000 iterations. lositan uses coalescent simulations to generate a null distribution of *F*_ST_ values, controlling for the corresponding expected heterozygosity value. The loci that lie outside the expected range under a neutral model of evolution are considered candidates for positive selection. We ran lositan using 50,000 simulations, a “neutral” forced mean *F*_ST_, confidence interval of 99%, and false discovery rate of 10%.

### 2.6 Clustering analyses

We first tested whether latitude was a good proxy for variation in environmental variables (Supporting Information). PCA of environmental variables showed that PC1 explained 88.5% of the variation, and was highly correlated with latitude (*r* = −0.97, *p* < 0.01, Fig. S1). Given the strong correlation between environmental variables and latitude, if murres from different colonies are adapted to conditions at their breeding grounds, grouping individuals based on genetic loci putatively under selection should show individuals from similar latitudes more closely associated than individuals breeding at more distant latitudes. We first investigated genetic differentiation between common and thick‐billed murres to test for hybrids. We then performed clustering analyses to test whether the loci putatively under selection grouped individual thick‐billed murres differently from the complete dataset (as in e.g., Milano et al., 2014; Keller et al., 2013). We analyzed two separate datasets—one that included all loci and one that included only outlier loci—using two different clustering methods. We used a discriminant analysis of principal components (DAPC; Jombart, Devillard, & Balloux, 2010) using the R package *Adegenet*, and the program Structure v.2.3.2 (Pritchard, Stephens, & Donnelly, 2000) to analyze each of the two datasets. DAPC partitions genetic variation into between‐group and within‐group components to identify groups for which the within‐group component of variation is minimized, and provides a graphical representation of the relatedness between groups. Additionally, DAPC is able to discriminate between complex population structure models, including hierarchical and stepping‐stone models (Jombart et al., 2010), and is therefore suitable to test for clinal variation in allele frequencies. We performed a DAPC and retained the number of principal components for each dataset that resulted in the greatest power of discrimination but avoided overfitting. structure is based on a Bayesian algorithm that clusters individuals to minimize deviations from Hardy–Weinberg equilibrium. We ran the program 20 times for each number of clusters (*K*) between 1 and 6 using prior population information, with a correlated allele frequency model, and a burn‐in of 50,000 generations followed by 500,000 generations. We used the same settings to analyze the dataset that included common murres. The most likely number of genetic populations was calculated using Evanno's Δ*K* (Evanno, Regnaut, & Goudet, 2005) with structure harvester (Earl & vonHoldt, 2012). We averaged membership probabilities among runs using clumpp v.1.1 (Jakobsson & Rosenberg, 2007) and displayed results using distruct v.1.1 (Rosenberg, 2004).

We adopted a randomization approach to test whether selecting the most differentiated SNPs among populations to investigate population structure at a fine scale is a circular approach, and whether outlier SNPs were informative of real population differentiation. We created a randomized genotype dataset by randomly reassigning population labels to each of the thick‐billed murre samples as outlined in Campagna, Gronau, Silveira, Siepel, and Lovette (2015). Then, we ran outlier analyses on this dataset using lositan and used outlier loci to investigate population structure in structure. We compared these results with results from the original dataset. If the approach to detect fine‐scale population structure using outlier loci was circular, we would expect to detect population structure based on outlier loci from both the original and the randomized dataset. Otherwise, if population structure was found only in the original dataset, outlier loci would represent real genetic differences among colonies.

### 2.7 Annotation of outlier loci

To investigate the identity of the outlier loci, we conducted BLAST (Altschul et al., 1990) searches of the sequences flanking each outlier SNP using the NCBI nucleotide collection database. The search was restricted to “Aves” and had an e‐value cutoff of 10^−10^. We also associated gene ontology (GO) annotation terms to all outlier SNPs using the Gene Ontology database (Carbon et al., 2009; Gene Ontology Consortium 2000). Enrichment analyses to compare representation of GO terms between all loci and only outliers were not performed due to the low number of outlier loci annotated using BLAST (see Results).

## 3 Results

### 3.1 Whole‐genome assembly

We obtained a total of 597 million raw paired‐end reads. The total assembly length was ~1.2 Gb, similar to other bird genomes (Jarvis et al., 2014), and average k‐mer coverage was ~40× (see Table S1 for assembly statistics). ray reported peak coverage at 20×, but it was mislead by the first peak of higher coverage (two peaks are expected because of heterozygous sites; Fig. S2). This is likely due to high levels of genomic heterozygosity (Zheng et al., 2013). Average base coverage was 57×.

### 3.2 RAD sequence data quality and processing

Seven of 100 samples were excluded at various stages of library preparation due to low DNA quality or concentration. We obtained 95,075,938 paired‐end 150 bp paired‐end reads, which represent more than 1 million paired reads per individual on average. Quality of reads was very high across libraries as indicated by an average phred quality score of 36. After demultiplexing, the number of reads was particularly low for three samples, which we then excluded from downstream analyses. The final dataset included 86 thick‐billed murres (Table 1) plus four common murres.

Processed reads aligned to the reference genome with an average overall rate of 93%. An average of 96% of mapped reads were retained after filtering for multiple hits and were used for downstream analyses. We built a catalog of 110,292 loci. After filtering in populations to include only loci that were polymorphic and present in at least 90% of the individuals from each of the five colonies, we obtained a dataset of 7032 SNPs distributed across 1248 loci, with an average depth of sequencing per SNP for each individual of 164×.

### 3.3 Genetic diversity

Most diversity indices had similar values among colonies (Table 2). The average major allele frequency (P) was 89.9%, frequency of polymorphic sites was 2.6%, and frequency of private alleles was 0.16%. No variation was found among colonies in observed heterozygosity (Table 2). Colonies differed significantly only in their inbreeding coefficients *F*_IS_, with Gannet having the highest value, and Coats the lowest, and the difference between these two colonies with extreme values was highly significant (one‐tailed *t*‐test, *p* = 0.0055). *F*_IS_ calculated across all loci and all colonies was significantly >0 (0.049, *p* ≪ 0.001, tested using genodive with 10,000 permutations). The global *F*_ST_ was not different from 0, with the highest *F*_ST_ for an individual SNP being 0.112 and most loci showing low levels of differentiation. Pairwise *F*_ST_ estimates between colonies were not different from 0 for any comparison (Table 3). However, locus‐specific *F*_ST_ estimates between pairs of colonies ranged up to 0.307. The low global *F*_ST_ value is not surprising given that this estimate represents the average divergence between populations relative to the total diversity sampled, populations are highly variable, and divergence is recent.

**Table 2:** Summary genetic statistics for all populations split into those calculated for only nucleotide positions that are polymorphic in at least one colony (top, “variant positions”), as well as all nucleotide positions across all restriction‐site‐associated DNA (RAD) sites regardless of whether they are polymorphic or fixed (bottom, “all positions”). These statistics include the average number of individuals genotyped at each locus (N), the number of variable sites unique to each population (private), the number of polymorphic (top) or total (bottom) nucleotide sites across the dataset (sites), percentage of polymorphic loci (% poly), the average frequency of the major allele (P), the average observed heterozygosity per locus (H_obs_), the average nucleotide diversity (π), and the average Wright's inbreeding coefficient (*F*_IS_)

	N	Private	Sites	% poly	P (%)	H_obs_	π	*F*_IS_
Variant positions								
Gannet	14.85	261	7005	66.78	89.9	0.144	0.155	0.034
Akpatok	18.63	314	7003	70.76	90.0	0.144	0.152	0.028
Coats	13.95	197	6998	64.98	90.0	0.149	0.153	0.016
Minarets	18.86	321	6994	71.39	89.8	0.146	0.154	0.023
Prince Leopold	18.92	379	6992	72.51	89.9	0.147	0.154	0.025
Average	17.05	294.4	6998.4	69.28	89.9	0.146	0.153	0.025
All positions								
Gannet	14.88	261	185277	2.52	99.6	0.005	0.006	0.0013
Akpatok	18.72	314	185273	2.67	99.6	0.005	0.006	0.0011
Coats	13.97	197	185268	2.45	99.6	0.006	0.006	0.0006
Minarets	18.90	321	185260	2.69	99.6	0.006	0.006	0.0009
Prince Leopold	18.52	379	185266	2.73	99.6	0.005	0.006	0.0009
Average	17.08	294.4	185268.8	2.62	99.6	0.006	0.006	0.0010

**Table 3:** Colony pairwise *F*_ST_ values when all loci are included (above diagonal) and when only outlier loci are included (below diagonal)

	Gannet	Akpatok	Coats	Minarets	PLI
Gannet	‐	0	0	0	0.001
Akpatok	0.045^a^	‐	0	0	0
Coats	0.044^a^	0.052^a^	‐	0.001	0.001
Minarets	0.038^a^	0.043^a^	0.054^a^	‐	0
Prince Leopold	0.035^a^	0.045^a^	0.044^a^	0.036^a^	‐

a Significantly different from 0 (*p *≪ 0.001).

The SNP dataset that included samples of both thick‐billed (*n* = 86) and common murres (*n* = 4) contained 2279 loci. Average *F*_ST_ between thick‐billed murres (*n* = 86) and common murres (*n* = 4) was high and significantly different from 0 (*F*_ST _= 0.435, *p* ≪ 0.001).

### 3.4 Outlier analyses

The filtering step based on minor allele frequencies eliminated all private alleles from the dataset, in that they all occurred at frequencies below 0.05. bayescan did not detect any outlier loci. The number of outlier SNPs identified by lositan ranged from 14 to 28 among runs. Akpatok showed the highest number of outliers when compared to Prince Leopold (28 SNPs) and Minarets (24 SNPs), respectively. The 137 SNPs identified among all runs were distributed among 111 loci, and 38 SNPs were indicated as potentially under positive selection in two or more pairwise comparisons, indicating either that populations may be diverging in parallel (Keller et al., 2013) or that these loci are located in area of low recombination (Burri et al., 2015).

With the dataset including only outliers from lositan, estimates of *F*_ST_ between colony pairs were between 0.035 and 0.054, and all estimates were significantly different from 0 (all *p* < 0.0001; Table 3).

### 3.5 Clustering analyses

structure assigned common and thick‐billed murres to separate clusters with 100% confidence even with the very conservative settings of an admixture model with no prior population information (Fig. S3). We observed the same results using DAPC (data not shown). We found no evidence of hybrids among murres included in our study.

**Figure 2:**
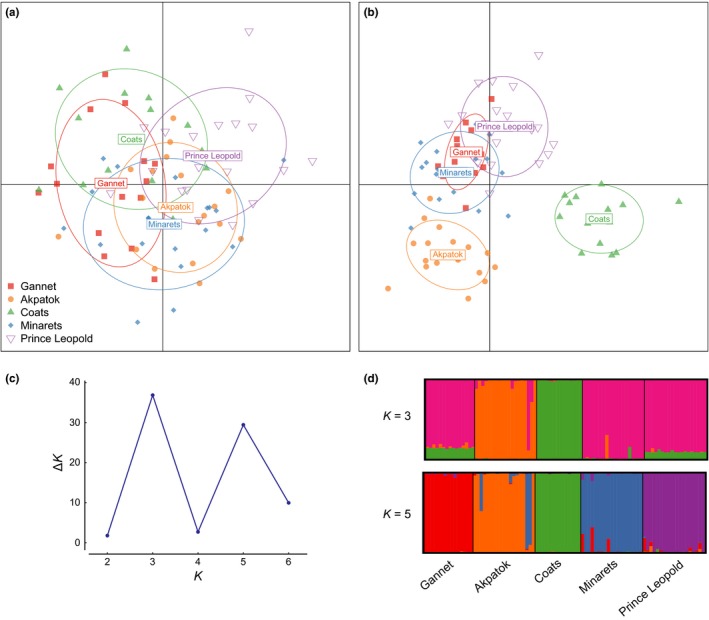
Plots of clustering analyses. (a) DAPC results for all loci and (b) only outliers; plots (c) and (d) are based on the ‘only outliers’ dataset; (c) plot showing Evanno's Δ*K* with two peaks corresponding to *K *=* *3 and 5; (d) barplots of STRUCTURE results for *K *=* *3, which received highest support from Evanno's Δ*K*

Results from structure and DAPC were highly concordant when applied to thick‐billed murres only. When all SNPs were included, structure failed to detect any population structure (data not shown). Similarly, the DAPC showed a high degree of overlap among colonies (Figure 2a). However, results from analyses that included only SNPs potentially under positive selection were very different. Both structure and DAPC grouped the southernmost colony, Gannet, with the two northernmost colonies, Minarets and Prince Leopold, whereas individuals from Coats and Akpatok clustered separately from each other and from any other colonies (Figure 2b,d). The graphical results were supported by Evanno's Δ*K*, which indicated highest support for three distinct populations (Figure 2c). Δ*K* also showed a peak at *K *=* *5, indicating that individuals are more similar within colonies than among colonies and suggesting hierarchical population structure (Figure 2b,d). Interestingly, two individuals from Akpatok were assigned to the Minarets population in structure and overlapped with individuals from Minarets in the DAPC as well. Because these two individuals could represent migrants from Minarets into Akpatok, we excluded them from the dataset and repeated the DAPC. When those two samples were removed from the analyses, Akpatok separated more strongly from the other colonies, making the DAPC results match the structure results (Fig. S4). lositan identified a total of 252 outlier SNPs from the randomized dataset, but both structure and the DAPC failed to detect population structure in this dataset, thus indicating that the apparent distinctiveness of the colonies is not an artifact of the analyses.

### 3.6 Annotation of outlier loci

Nine of the 111 outlier loci were successfully annotated. Of these, three loci were identified as unknown genes, genes of unknown function, or microsatellite sequences. Six loci were assigned GO terms, which included immune response, synaptic transmission, cellular processes, and membrane proteins (Table S3).

## 4 Discussion

### 4.1 Genetic structure and inbreeding

Overall, we found no differentiation among thick‐billed murres from different colonies along the Atlantic Canada coast based on the entire dataset (2220 SNPs). The presence of even subtle population structure did not receive support from any of our analyses run on the entire dataset, including estimates of colony pairwise *F*_ST_ (Table 3), and clustering analyses based on either structure or DAPC (Figure 2a). Note that *F*_ST_ can be deflated by high genetic diversity, which is observed in our dataset (see details below). These results are consistent with previous analyses of mitochondrial and microsatellite data, which also failed to detect population structure in the Atlantic Ocean (Birt‐Friesen et al., 1992; Tigano et al., 2015). Lack of population structure in marine organisms, including seabirds, is often observed due to apparent lack of barriers to gene flow and/or large population sizes (Friesen, 2015; Gagnaire et al., 2015). In some cases, the use of a high number of markers can increase resolution: In the black‐footed albatross (*Phoebastria nigripes*) for instance, genotyping of thousands of genomewide markers revealed weak but significant population structure among colonies in Japan and Hawaii, where previous results from traditional genetic markers were contradictory (Dierickx, Shultz, Sato, Hiraoka, & Edwards, 2015). However, similar to our results, in other studies where mtDNA and microsatellite data failed to detect population structure, the use of thousands of markers did not detect genetic differentiation, even among species (e.g., Campagna et al., 2015).

Genetic statistics were very similar among colonies, with the exception of *F*_IS_ (Table 2). In population genetics, positive values of *F*_IS_ indicate that mates are more closely related than random individuals within a population. While all other *F*‐statistics were similar among colonies in the present study, *F*_IS_ was significantly >0 in all colonies and was highest at Gannet. Positive values of *F*_IS_ also suggest cryptic population structure. If within‐colony relatedness of thick‐billed murres decreases with increasing colony size (Friesen, Montevecchi, Gaston, Barrett, & Davidson, 1996; Ibarguchi, Gaston, & Friesen, 2011), then the high inbreeding coefficient at Gannet could be explained by its population size: two to three orders of magnitude smaller than other colonies included in this study (Gannet < 1000 breeding pairs, Coats ~ 30,000, and Akpatok, Minarets, and Prince Leopold >100,000 Gaston, Mallory, & Gilchrist, 2012).

### 4.2 Outlier analyses: selection or demography?

Outlier analyses can be powerful tools to detect loci that are under selection, especially when levels of background genomic differentiation are low (e.g., Hess et al., 2013; Keller et al., 2013; Milano et al., 2014). However, several factors can limit the power of these methods or confound the interpretation of results (Mita et al., 2013; Edwards et al., 2015; Lotterhos & Whitlock, 2014; Narum & Hess, 2011). bayescan did not detect outlier loci in our dataset. bayescan could lose power with low number of populations and/or weak selection. The strength of selection was not known *a priori*, but several results indicate that local selection might be weak: We did not identify fixed differences between colonies; private alleles represented only a small fraction of the variant sites and occurred at low frequency (all below 0.05); locus‐specific *F*_ST_ between colonies never exceeded 0.307; and although structuring based on the outlier loci identified with lositan was indicated by structure when we used the admixture model, clear separation between colonies was evident only when we used the no admixture model, which is more powerful at detecting weak structure. The genomic architecture of traits under selection can also give the appearance of weak selection. If a trait is polygenic, the selection coefficient *s* will be divided among the SNPs that contribute to that trait, so that the more polygenic a trait is, the lower *s* will be on each SNP, thereby hindering the ability to identify loci under selection (Yeaman, 2015).

lositan provides a more robust method than bayescan when the number of populations screened is low, or when populations deviate from the island model of migration on which lositan is based (Beaumont & Nichols, 1996; Mita et al., 2013; Narum & Hess, 2011). We therefore used the outlier loci identified from lositan to investigate local adaptation. Analyses of environmental variation during the breeding period showed that temperature, precipitation, sea ice coverage, and timing of ice freeze‐up and breakup vary greatly and clinally among colonies. Presumably, if birds were locally adapted to their breeding grounds, birds from colonies that experience more similar conditions should be more similar at loci putatively under selection. structure analyses based only on outlier loci found the highest statistical support for three genetic populations: (1) a group that included samples from Gannet, Minarets, and Prince Leopold; (2) Coats; and (3) Akpatok. Similarly, birds from Gannet, Minarets, and Prince Leopold clustered together in the DAPC, whereas birds from Coats and Akpatok clustered separately from the rest of the colonies. The fact that the southernmost colony (Gannet), which is climatically the most different from the rest of the colonies, grouped together with the two northernmost colonies (Minarets and Prince Leopold) in both clustering analyses rejects our hypothesis of local adaptation to the breeding grounds along a latitudinal cline.

We tested whether random processes drove the pattern of differentiation among colonies by analyzing a randomized dataset. For example, in southern capuchino seedeaters (genus *Sporophila*), this approach revealed that putative differentiated loci were not really informative of differentiation among species because the same pattern could be obtained from a randomized dataset (Campagna et al., 2015). In our case, however, outlier loci identified from the randomized dataset failed to indicate any structuring. These results not only support that the pattern of differentiation based on the original dataset was not random, but also that analyzing outlier loci to investigate fine patterns of differentiation is a suitable approach if accompanied by rigorous randomization tests.

The pattern of differentiation observed at outlier loci could be driven by selection or demographic processed during the non‐breeding period. Results from structure and the DAPC are consistent with nonbreeding distributions of thick‐billed murres from different colonies, as inferred from data from geolocators (McFarlane Tranquilla et al., 2013). Non‐breeding distributions of birds from Prince Leopold, Minarets, and Gannet overlap considerably off the coast of Newfoundland, whereas distributions of non‐breeding birds from Coats overlap only with birds from Prince Leopold, and only by ~20% (McFarlane Tranquilla et al., 2013). If we also consider the temporal pattern of migration, birds from Coats and Prince Leopold overlap briefly only in January (Gaston et al., 2011; McFarlane Tranquilla et al., 2013). The birds from Coats differ from the rest of the birds included in this study in that they spend nearly eight months in Hudson Bay — oceanographically a very different system from Davis Strait and the Labrador and Newfoundland shelves, where birds from other colonies spend most of their non‐breeding period (Gaston et al., 2011). Unfortunately no data on non‐breeding distributions are available for birds from Akpatok. Environmental conditions during the winter might be a strong selective force promoting adaptation in this and other seabirds. In thick‐billed murres from Coats, while breeding success was associated with environmental conditions near the colony during the breeding period, annual adult survival was most correlated with environmental conditions on the non‐breeding grounds (Smith & Gaston, 2012). Additionally, energy expenditure analyses indicate that energy requirements increase during the winter in thick‐billed murres and little auks (*Alle alle*), a smaller Alcid seabird, mainly due to air temperature and wind speed (Fort, Porter, & Grémillet, 2009). These results combined suggest that although environmental conditions at the breeding grounds affect the survival of chicks, selection during the non‐breeding period might be stronger than during the breeding period, which would explain the pattern of differentiation at outlier loci. However, the environmental conditions that thick‐billed murres experience during the nonbreeding period vary greatly in time and space (McFarlane Tranquilla et al., 2015), and pinpointing the selective agent during this period would be difficult. In general, thick‐billed murres seem to be able to cope with a variety of environmental conditions (McFarlane Tranquilla et al., 2015). The ability to adjust to a variable environment could be enabled by phenotypic plasticity, high levels of standing genetic variation, or both (Crispo, 2008; Pritchard, Pickrell, & Coop, 2010). We did observe high levels of standing genetic variation (although we do not know whether this variation has adaptive value), but levels of phenotypic plasticity are virtually unknown in this species. While spatially varying selection can promote local adaptation (Tigano & Friesen, 2016), temporal variation in selection can favor generalist genotypes and phenotypic plasticity over local adaptation (Kawecki & Ebert, 2004).

However, the fact that birds from colonies that migrate to the same areas are more genetically similar could be explained by gene flow due to higher demographic connectivity at the non‐breeding grounds among these birds. Although thick‐billed murres are highly philopatric, immature birds may migrate back to the breeding grounds following birds from a different colony rather than the natal one. This would explain the two samples from Akpatok that were assigned to Minarets, and the clustering of birds from Gannet, Minarets, and Prince Leopold, whose non‐breeding grounds overlap considerably. The role of gene flow is also supported by the observation that birds from Coats, which overlap minimally with birds from other colonies at the non‐breeding grounds (Gaston et al., 2011), differ the most from the rest of the birds included in this study. These considerations indicate that either even a little gene flow is sufficient to homogenize genetic variation and disrupt local adaptation if selection is weak, or band returns underestimate the extent of current gene flow among colonies (Coulson, 2016).

The recolonization history of thick‐billed murres also corresponds to the pattern of differentiation among colonies. The timing of ice retreat following the last glacial maximum at the five locations included in this study matches their genetic relationship (Dyke, 2004): ice retreated first along the cost, that is, from Gannet and Minarets, which overlaps the most in the DAPC, followed by Prince Leopold, which overlaps partially with Gannet and Minarets. Ice retreat in the Hudson Bay occurred later, from east to west, at Akpatok first and then at Coats.

Evolutionary change is the result of the interaction between selection, gene flow, and genetic drift. Population genetic theory predicts that genetic drift is reduced in organisms characterized by large effective population size and high genetic variation. Thick‐billed murres feature both these characteristics: Each of the colonies screened in this study contains hundreds to hundreds of thousands breeding pairs (Gaston et al., 2012); genetic variation is high, as indicated by the high heterozygosity in the whole‐genome sequence, high frequency of polymorphic sites, absence of fixed differences among colonies, and previous analyses of neutral markers (Tigano et al., 2015). Thick‐billed murres show higher nucleotide diversity than most species for which these data are available (Shultz, Baker, Hill, Nolan, & Edwards, 2016 and references therein). Compared to black‐footed albatrosses, for example, thick‐billed murres have higher nucleotide diversity and number of polymorphic sites by an order of magnitude (Dierickx et al., 2015). Even though the role of genetic drift is presumably minimal in our study system, selection and gene flow can generate the same pattern. Genomic and migration data from additional colonies could help testing the relative roles of drift, selection, and gene flow.

Our results add to the increasing evidence that differentiation can vary along the genome. Whole‐genome analyses are starting to show that this heterogeneous genomic landscape is due not only to locally varying gene flow‐selection balance but also to varying recombination rates across the genome, based on the observation that areas of differentiation seem often to be localized in areas of low recombination (Burri et al., 2015; Tigano & Friesen, 2016). In the future, higher genomic coverage will allow us to test the role of recombination in maintaining few differentiated loci against a homogeneous genomic background in this study system.

Due to the small proportion (~5%) of outlier loci assigned with GO terms, neither enrichment analyses nor any other inference regarding the biological function of outlier loci was possible. However, the low assignment rate could indicate that (1) outlier loci may be located in regulatory regions or other regions of functional importance that are under selection but are not annotated; (2) several genes of small effect may have not been detected due to low sensitivity of outlier detection methods (Yeaman, 2015); (3) some of the outlier loci are false positives with respect to selection, and the results of clustering analyses indicate demographic connectivity rather than selection; or (4) other genes potentially involved in local adaptation may not have been sequenced.

Although lack of population structure in seabirds and other marine organisms is very common (e.g., Friesen, 2015), the use of a limited number (<100) of highly divergent loci can help identify population structure (Funk, McKay, Hohenlohe, & Allendorf, 2012). For example, this approach successfully identified the geographic origin for several species of fish of economic importance (Hess, Matala, & Narum, 2011; Nielsen, Hemmer‐Hansen, Larsen, & Bekkevold, 2009; Nielsen et al., 2012), and the strength of migratory connectivity in a passerine (Ruegg et al., 2014). Although our data are limited to five colonies and more genetic and spatial data would be necessary for a robust test, our study represents a first indication for an association between genetic differentiation and nonbreeding distribution in a natural population of birds.

## 5 Conclusions

Lack of population structure despite high philopatry is observed in many other seabird species (reviewed in Friesen, 2015). However, we observed a clear and consistent pattern of differentiation among colonies across analyses based on outlier loci: Outlier loci do not vary with latitude of breeding colonies as hypothesized, but rather seem to group birds from different colonies according to their nonbreeding distribution, although it is not clear whether this association is due to selection and/or demographic connectivity. Our study highlighted the inherent limitations of outlier analyses in detecting signatures of local adaptation in natural populations, especially when the same pattern of differentiation can be driven by multiple factors such as gene flow, genetic drift, recombination rate, and demographic history. Nonetheless, we showed that if we consider the demographic histories and evolutionary scenarios of the focal species, the limitations of the methods to detect outlier loci, and all alternative hypotheses explaining differentiation patterns, outlier analyses could also be used to investigate demographic processes in populations of species that are demographically independent but not yet not genetically differentiated (Gagnaire et al., 2015). Although with five colonies we did not have enough power for more rigorous tests (e.g., genotype–environment association tests), the results from this study are promising in sight of future studies including more colonies from throughout the Atlantic. Ultimately, a candidate gene approach or whole‐genome resequencing to detect loci under selection may be required to test hypotheses on adaptation in this and other species displaying only weak signals of spatial variation (Edwards et al., 2015).

## Conflict of Interest

None declared.

## Author Contributions

A.T., A.J.S, S.V.E., and V.L.F. designed research. G.J.R facilitated funding and provided expertise on the study system and its environment. A.T. and A.J.S collected data; A.T. analyzed the data with the help of A.J.S. and wrote the manuscript with the help of all co‐authors.

## Data Accessibility

The thick‐billed genome assembly and input files for all the analyses conducted in this study are available from the Dryad Digital Repository: doi:10.5061/dryad.7182c.
