# Supplementary material for: Outlier analyses to test for local adaptation to breeding grounds in a migratory arctic seabird
Source: Ecol Evol. 2017 Mar 12;7(7):2370–81. doi: 10.1002/ece3.2819 (PMC5383466; doi:10.1002/ece3.2819)
Supplement: Supplementary file 1 [file ECE3-7-2370-s001.docx]

**Supporting Information**

*Environmental differences among breeding colonies*

We analyzed climatic variables to quantify environmental differences among colonies along the latitudinal cline. We obtained ice break-up and freeze-up dates, weekly median ice concentration for June 4-July 2, and weekly median of ice concentration when present for July 2-July 30 from the Canadian Ice Service database (data for years 1981-2000; http://iceweb1.cis.ec.gc.ca/). We extracted WorldClim variables (data for years 1950-2000 at ~1 km resolution; Hijmans *et al.*, 2005) for each colony using the website http://dataportal-senckenberg.de/dataExtractTool. We downloaded data on monthly mean, minimum and maximum temperature during the breeding period (May-September) and six BIOCLIM variables, including annual mean temperature (BIO1), mean diurnal temperature range (BIO2), maximum temperature at warmest month (BIO5), mean temperature of warmest quarter (BIO10), annual precipitation (BIO12) and precipitation of warmest quarter (BIO18). Because WorldClim data cover only landmasses and coverage on coastal locations can be incomplete, we approximated spatial coordinates by moving all locations ~1 km inland using Google Earth 7.1.2.2041 (2013). We performed a principal components analysis (PCA) including all environmental variables described above and tested whether the principal components (PC) explaining most variation correlated with latitude.

Fig. S1 Plot showing the relationship between latitude and PC1 obtained from a PCA of environmental variables.

Fig. S2 Thirty-one k-mer depth of coverage distribution of whole-genome reads. Two peaks at ~20 and ~40 were identified. K-mer, a unique sequence of k nucleotides long.

Fig. S3 STRUCTURE plot representing genetic structure between common murres and thick-billed murres. Individuals are assigned to the respective species with 100% confidence.

Fig. S4 DAPC plot for only outliers loci after excluding two individuals from Akpatok that were originally assigned to Baffin.


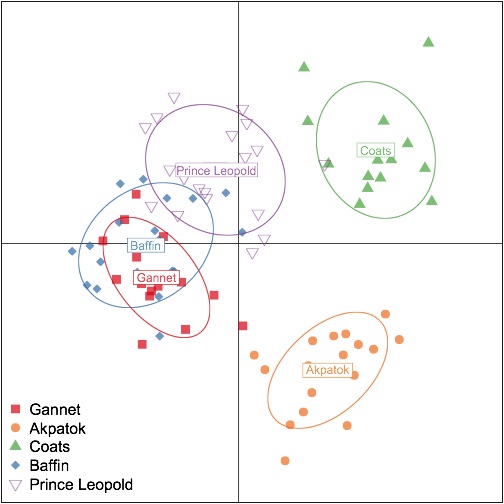


Table S1 Environmental data included in the PCA for each of the five colonies.

|  | Colony | | | | |
| --- | --- | --- | --- | --- | --- |
| Environmental variable | Gannet | Akpatok | Coats | Minarets | Prince Leopold |
| Ice-free days | 232 | 141 | 140 | 98 | 28 |
| Average ice concentration when present in July (percentage) | 31% | 45% | 48% | 58% | 73% |
| Average ice concentration in June (percentage) | 0% | 69% | 78% | 87% | 95% |
| Mean T in May (°C) | 2.1 | -2.1 | -5.3 | -4.8 | -9.5 |
| Mean T in June (°C) | 6.7 | 3.4 | 2.9 | 1.6 | 1.3 |
| Mean T in July (°C) | 10.6 | 7.3 | 9.0 | 5.9 | 5.7 |
| Mean T in August (°C) | 10.7 | 7.2 | 7.7 | 5.1 | 3.9 |
| Mean T in September (°C) | 7.5 | 3.4 | 2.0 | 5.0 | -3.6 |
| Min T in May (°C) | -1.3 | -5.2 | -9.5 | -8.4 | -13.0 |
| Min T in June (°C) | 2.3 | 0 | -9.0 | -1.3 | -1.4 |
| Min T in July (°C) | 5.9 | 3.1 | 3.9 | 2.4 | 2.4 |
| Min T in August (°C) | 6.5 | 3.4 | 3.1 | 1.9 | 1.1 |
| Min T in September (°C) | 4.1 | 4.0 | -1.4 | -2.1 | -5.9 |
| Max T in May (°C) | 5.6 | 1.0 | -1.0 | -1.1 | -5.9 |
| Max T in June (°C) | 11.1 | 6.8 | 6.7 | 4.6 | 4.0 |
| Max T in July (°C) | 15.4 | 11.6 | 14.1 | 9.4 | 9.1 |
| Max T in August (°C) | 14.9 | 11.0 | 12.3 | 8.4 | 6.8 |
| Max T in September (°C) | 11.0 | 6.5 | 5.5 | 3.2 | -1.3 |
| Precipitation in May (mm) | 60 | 20 | 17 | 38 | 7 |
| Precipitation in June (mm) | 81 | 36 | 27 | 29 | 10 |
| Precipitation in July (mm) | 85 | 49 | 40 | 32 | 20 |
| Precipitation in August (mm) | 86 | 57 | 48 | 41 | 26 |
| Precipitation in September (mm) | 89 | 44 | 39 | 53 | 20 |
| Annual mean temperature (bio1,°C) | -0.3 | -6.6 | -10.1 | -9.2 | -15.5 |
| Mean diurnal temperature range (bio2,°C) | 7.7 | 7.1 | 9.1 | 7.4 | 6.8 |
| Max T at warmest month (bio5,°C) | 15.4 | 11.6 | 14.1 | 9.4 | 9.1 |
| Mean T at warmest quarter (bio10,°C) | 9.6 | 5.9 | 6.5 | 4.2 | 3.6 |
| Precipitation at warmest quarter (bio18, mm) | 260 | 142 | 115 | 102 | 56 |
| Annual precipitation  (bio12, mm) | 957 | 370 | 283 | 455 | 119 |

Table S2 Summary of genome assembly statistics.

|  | Contigs | Scaffolds |
| --- | --- | --- |
| Number greater than 5 kb | 65890 | 42268 |
| Number greater than 10 kb | 34526 | 25121 |
| Number greater than 20 kb | 12813 | 14737 |
| Number greater than 40 kb | 3193 | 6647 |
| Number greater than 80 kb | 469 | 2137 |
| Number greater than 160 kb | 39 | 512 |
| Number greater than 320 kb | 0 | 79 |
| Number greater than 640 kb | 0 | 8 |
| Number greater than 1 Mb | 0 | 1 |
| Total number | 272060 | 210291 |
| Total bases | 1218121286 | 1240211403 |
| Minimum length | 123 | 123 |
| Maximum length | 282069 | 1267403 |
| N25 | 29018 | 78463 |
| N50 | 14218 | 32998 |
| N75 | 6461 | 9958 |
| N90 | 2663 | 3187 |
| GC content | 40.95 | |
| Ns content | 5.2 | |

Table S3 BLAST results of outlier loci.

| Locus ID | Gene identity | Gene Ontology |
| --- | --- | --- |
| 272 | mRNA for hypothetical protein | - |
| 1287 | Cytidine monophosphate kinase 1, cytosolic | Biosynthetic process |
| 4485 | Transmembrane protein 196 | Integral component of membrane |
| 6226 | Microsatellite sequence | - |
| 6982 | Deletion in malignant brain tumor 1 | Response to estrogen  Inner cell mass cell proliferation  Protein transport  Tissue regeneration |
| 8235 | Chloride channel CLIC-like 1 | Chloride transmembrane transport |
| 9271 | Gap junction protein delta 2 | Cell-cell signaling  Synaptic transmission  Visual perception |
| 14225 | MHC class I antigen | Immune response  Antigen processing and presentation of peptide antigen via MHC class I |
| 18236 | Unknown protein | - |

Last file loaded on 2015-04-14; AmiGO 2 v.2.1.4

**References**

Google Earth 7.1.2.2041. 2013. Arctic region [cited 2016 March 1]. Available from: http://www.google.com/earth.

Hijmans, R.J., Cameron, S.E., Parra, J.L., Jones, P.G., & Jarvis, A. 2005. Very high resolution interpolated climate surfaces for global land areas. *Int. J. Climatol.* **25**: 1965-1978.
